# Lung cancer and passive smoking: reconciling the biochemical and epidemiological approaches.

**DOI:** 10.1038/bjc.1992.341

**Published:** 1992-10

**Authors:** R. L. Tweedie, K. L. Mengersen

**Affiliations:** Department of Statistics, Colorado State University, Fort Collins 80523.

## Abstract

The accurate determination of exposure to environmental tobacco smoke is notoriously difficult. There have been to date two approaches to determining this exposure in the study of association of passive smoking and lung cancer: the biochemical approach, using cotinine in the main as a marker, and the epidemiological approach. Typically results of the former have yielded much lower relative risk than the latter, and have tended to be ignored in favour of the latter, although there has been considerable debate as to the logical basis for this. We settle this question by showing that, using the epidemiologically based meta-analysis technique of Wald et al. (1986), and misclassification models in the EPA Draft Review (1990), one arrives using all current studies at a result which is virtually identical with the biochemically-based conclusions of Darby and Pike (1988) or Repace and Lowry (1990). The conduct of this meta-analysis itself raises a number of important methodological questions, including the validity of inclusion of studies, the use of estimates adjusted for covariates, and the statistical significance of estimates based on meta-analysis of the epidemiological data. The best estimate of relative risk from spousal smoking is shown to be approximately 1.05-1.10, based on either of these approaches; but it is suggested that considerable extra work is needed to establish whether this is significantly raised.


*Br. J. Cancer* (1992), **66**, 700–705 © Macmillan Press Ltd., 1992

### Lung cancer and passive smoking: reconciling the biochemical and epidemiological approaches

R.L. Tweedie[1] & K.L. Mengersen[2]

[1]*Department of Statistics, Colorado State University, Fort Collins, Colorado 80523, USA;* [2]*Department of Mathematics and Computing, University of Central Queensland, Rockhampton 4702, Australia.*

**Summary** The accurate determination of exposure to environmental tobacco smoke is notoriously difficult. There have been to date two approaches to determining this exposure in the study of association of passive smoking and lung cancer: the biochemical approach, using cotinine in the main as a marker, and the epidemiological approach. Typically results of the former have yielded much lower relative risk than the latter, and have tended to be ignored in favour of the latter, although there has been considerable debate as to the logical basis for this. We settle this question by showing that, using the epidemiologically based meta-analysis technique of Wald *et al.* (1986), and misclassification models in the EPA Draft Review (1990), one arrives using all current studies at a result which is virtually identical with the biochemically-based conclusions of Darby and Pike (1988) or Repace and Lowry (1990). The conduct of this meta-analysis itself raises a number of important methodological questions, including the validity of inclusion of studies, the use of estimates adjusted for covariates, and the statistical significance of estimates based on meta-analysis of the epidemiological data. The best estimate of relative risk from spousal smoking is shown to be approximately 1.05–1.10, based on either of these approaches; but it is suggested that considerable extra work is needed to establish whether this is significantly raised.

Exposure to 'passive smoking', or environmental tobacco smoke (ETS) is far from easy to measure accurately (Lee, 1988), since it is not a direct consequence of actions of the 'exposed' subjects. This means that standard research methods have been particularly difficult to use in considering the level of association between ETS and occurrence of diseases, even when strong levels of association have been reported between active smoking and those diseases.

In the 1970's it was accepted that either the levels of exposure or the types of exposure to ETS were such that no significant association between ETS and lung cancer existed. In the last decade, however, there have been well over 20 published epidemiological studies which have sought to investigate the association between exposure to ETS and lung cancer. This epidemiological approach (see Sections 3, 4) categorises exposure to ETS in terms of the smoking of spouses (except for Kabat, 1990 who uses home exposure) when the subject evaluated is a never-smoker, and then adjusts for now well-established differential sampling biases. The outcome assessed in these papers is usually occurrence of general lung cancer, although several concentrate on or are dominated by one specific form of this disease (e.g. Trichopoulos *et al.* (1981, 1983), who exclude adenocarcinoma, or Garfinkel (1985) whose results conversely are dominated by adenocarcinoma); but in general all types are considered.

Individual studies usually report overall relative risks or odds ratios and assess whether these are significantly raised from unity: that is, whether there is a significantly increased risk of lung cancer associated with ETS. In most individual studies, relative risks have not been significantly raised, but this could be due to the small size of the studies; and over the past 5 years there have been various evaluations using 'meta-analysis' of the overall risk of lung cancer following exposure to ETS. The two best known are the NRC Report (1986) and the paper by Wald *et al.* (1986). There are marked similarities in their evaluations, in that both calculate a 'combined relative risk', estimate the extent of bias due to differential misclassification of smokers and background exposure problems, and evaluate significance from the confidence levels established from the meta-analysis adjusted for misclassification, as in Sections 3 and 4 below. The EPA Draft Review (1990) and the recent review paper by Repace and Lowry (1990) also follow this pattern. These meta-analyses typically show a combined excess risk of around 0.3–0.5 for exposure to such spousal smoking, but this has been criticised largely because of the difficulties in accurate determination of exposure status (Lee, 1988; Uberla, 1987).

Simultaneously with the epidemiological studies, there has been a second and more direct approach to the problem of estimating levels of ETS exposure. The biochemical marker approach (see Section 2) attempts to measure takeup of nicotine derivatives, converts this to a 'cigarette equivalent' figure, and estimates a relative risk based on extrapolating the observed relative risk for active smoking.

Both the NRC Report (1986) and the EPA Draft Review (1990) review this biochemical 'cigarette-equivalent' approach, as does the review by Repace and Lowry (1990). All find substantially lower results for the excess risk using this biochemical marker method, of the order of 0.03–0.10, and this is supported by a similar model-based approach of Darby and Pike (1988).

This discrepancy clearly presents some methodological problems as to which level of association is correct, and by implication which approach (if either) to determining exposure is reliable. This has engendered considerable recent and rather inconclusive debate (see Darby & Pike, 1988; Wald *et al.*, 1990; 1991*a,b*; Lee *et al.*, 1991*a,b*).

Since the reviews cited above have been carried out, results from a number of further epidemiological studies have been published. These enable a revised calculation of the risk from the epidemiological data, and one purpose of our paper is to carry out such a revised assessment.

We show that after the now-standard 'Wald adjustment' for differential misclassification, the two methods can be reconciled to provide an estimate of the relative risk of lung cancer associated with exposure to ETS, with a best current estimate of around 1.07.

The conduct of the meta-analysis itself raises a number of questions which are important in the use of this increasingly popular tool for combining otherwise small and insignificant studies, and these are discussed in relation to establishing this estimate and its significance level.

Correspondence: R.L. Tweedie.
Received 4 July 1991; and in revised form 24 April 1992.

### 2. Cotinine, exposure to ETS and 'cigarette equivalents'

Cotinine is a metabolite of nicotine which is widely used as a marker of exposure to tobacco smoke.

The NRC Report (1986, p. 226) states that 'urinary cotinine is at present the best marker of tobacco smoke intake for passive smoking dosimetry'. Darby and Pike (1988) affirm that cotinine has proved 'most useful in assessing average daily exposure to tobacco smoke' and Repace and Lowry (1990) agree that 'nicotine and cotinine are the best markers (of exposure to ETS) currently available'.

The technique for assessing overall risk for exposure to ETS from estimated active smoking risks using such a marker uses an extrapolation argument. We assume that the relative risk of lung cancer occurring for active smokers is estimated to be $RR_A = 1 + E_A$, where $E_A$ is the known excess risk for active smokers, and that we wish to estimate the relative risk $RR_{ETS} = (1 + E_{ETS})$ of exposure to ETS, where $E_{ETS}$ denotes excess risk of this exposure.

Suppose the ratio of cotinine observed in the population exposed to ETS when compared to cotinine observed in active smokers is $\rho$. Since it has been estimated (cf Darby and Pike (1988), NRC Report (1986)) that the half life of cotinine is about 50% longer in active smokers than in non-smokers, if we assume that the continine ratio is directly linearly linked to the ratio of excess risks, we get

$$\rho = 1.5\ E_{ETS}/E_A$$

There are then two parameters which must be estimated to conclude this argument, namely $\rho$ and $E_{ETS}$, and both have been subjects of some debate.

The data of Wald and Ritchie (1984) has been used by, for example, Wald *et al.* (1986) to provide an estimate of $\rho$ of around 1% for the ratio of cotinine observed in the population exposed to ETS when compared to cotinine observed in active smokers, whilst Jarvis *et al.* (1984) provide an estimate of the ratio $\rho$ of around 0.6% to 0.8%. Lee (1991*a*) argues that the higher figure of 1% is biased upwards by possible inclusion of smokers; Wald *et al.* (1991*a*) argue that the lower value of 0.6% to 0.8% is invalid due to problems of definition and censoring of high values.

However, assuming that this range of 0.6–1% is at least reasonable gives a value of $E_{ETS}/E_A$ of 0.4% to 0.66%.

The excess risk $E_A$ for active female smokers is variously taken as seven (Wald *et al.*, (1986) and the NRC Report (1986)), or 11 (EPA Draft Review (1990)). Wald *et al.* (1991*a*) argue that since the level of active smoking is by the husband, the value of 11 for the excess risk of currently active male smoking is relevant, although again Lee (1991*b*) disputes this, and suggests that 7.3 is appropriate, being the excess for ever-smoking males rather than currently active smokers.

The lowest of these combinations of estimates yield a value of $RR_{ETS} = 1.028$, whilst the highest estimates give $RR_{ETS} = 1.08$ for the relative risk of those exposed to environmental tobacco smoke.

This range is also supported by other approaches using cotinine which have been used to evaluate relative risk levels.

Darby and Pike (1988), in a detailed model of lung cancer evolution, show that, if the dosage taken up by those exposed to ETS is equivalent on average to active smoking of 0.1 cigarettes per day, then the relative risk for those exposed from age 20 to age 65 extrapolated from active smoking estimates will be 1.06 (see Table III of Darby and Pike (1988)). Darby and Pike (1988) themselves state that relative cotinine levels should indicate, from their detailed model, an estimate for excess risk of around 'one seventeenth to one fifth' of 0.5, i.e. a value of $RR_{ETS}$ of around 1.03 to 1.10.

The value of 0.1 'cigarette equivalents' here is at the high end of the range put forward by Darby and Pike (1988, p. 830), although it is at the low end of the range suggested by Vutuc (1984). Repace and Lowry (1990, p. 29) assert as reasonable an assumption of uptake of nicotine by those exposed to ETS of 0.5–1% of the uptake by active smokers. This gives effectively the same level of 'cigarette equivalent' as that used by Darby and Pike (1988).

Overall, the use of the cotinine argument is agreed by all these authors to lead to a point estimate for the relative risk to females of lung cancer associated with exposure to ETS of around 1.03–1.10, with a 'best' value around $RR_{ETS}$ of 1.06 being consistent with several different sources of available published data.

Clearly there are questions open to dispute and perhaps in need of further and rather better data. We note only that although the suggested range of excess relative risks is wide in relative terms (with a maximum over twice the minimum), for all practical purposes 1.03–1.10 is a narrow set of values. The real methodological problem is that this range of values is much lower than that for the combined epidemiological analyses of the NRC Report or the EPA Draft Review (1990), which are around 1.3–1.5 after adjustment for well-established differential biases. In the next section we follow the Wald *et al.* (1986) meta-analysis and misclassification adjustment approach to reevaluate these estimates with the inclusion of new studies, and show that in fact they can be reconciled with these dosimetric values.

### 3. The meta-analysis approach for epidemiological data

### *Combined estimate of relative risk*

In any one study, data may be insufficient for accurate estimates of relative risks to be made. This may be evidenced by a statistically insignificant result, which may be due not to lack of an association but to low power of the tests used and low numbers in the study itself. The meta-analysis approach allows for results to be 'pooled' over a number of comparable studies, in order to gain a more accurate estimate of the real relationship to be made. There are different techniques for such pooling, but the concept has been used widely in recent assessment of the *overall* risk of passive smoking.

The use of this technique for epidemiological data was pioneered in the paper by Wald *et al.* (1986), following its development for clinical studies by Yusuf *et al.* (1985), and the technique has been adopted in the NRC Report (1986) and the EPA Draft Review (1990).

Since the NRC Report (1986) and the Wald *et al.* (1986) paper have been published, a number of further studies of the relationship between exposure to ETS and the risk of lung cancer have been published. Many of these further studies are included in the EPA Draft Review (1990), although Varela (1987) is not included in the EPA Draft Review meta-analysis, and Wu-Williams *et al.* (1990), Sobue *et al.* (1990), Kabat (1990), Kalandidi *et al.* (1990), Liu *et al.* (1991) and Fontham *et al.* (1991) have appeared since the preparation of the EPA Draft Review (1990).

Table I gives the relative risks and associated confidence intervals, calculated (except for Varela (1987)) using the logit method, of all case-control studies on females published to date. (The numbers of males who are never-smokers and who contract lung cancer constitute a very small study population. Addition of those investigations which report on males does not materially change the results in Table I). The Varela (1987) estimate is itself a combined estimate, using the variance-weighted method of Wald *et al.* (1986), of the estimates in Table II of Varela (1987).

### *Adjusting for misclassification and bias*

The paper of Wald *et al.* (1986) developed a technique for estimating the effect of differential bias introduced by the misclassification of smokers as non-smokers. The NRC Report (1986), and the EPA Draft Review (1990) followed this methodology, with the EPA Draft Review modifying it somewhat to incorporate effects of misclassification of ex-smokers directly.

The key observation of Wald *et al.* (1986) is that, because smokers tend to marry smokers, if a study contains subjects who are assessed as non-smokers when they are not, they are

**Table I** Known odds ratios for studies of female non-smokers exposed to ETS

| Source | *Odds ratio* | *Upper 95% CI* | *Lower 95% CI* |
|---|---|---|---|
| Akiba *et al.* (1986) | 1.52 | 2.63 | 0.87 |
| Brownson *et al.* (1987) | 1.52 | 5.96 | 0.39 |
| Buffler *et al.* (1984) | 0.80 | 1.90 | 0.34 |
| Chan & Fung (1982) | 0.75 | 1.30 | 0.43 |
| Correa *et al.* (1983) | 2.07 | 5.25 | 0.81 |
| Fontham *et al.* (1991) | 1.32 | 1.68 | 1.03 |
| Gao *et al.* (1987) | 1.19 | 1.73 | 0.82 |
| Garfinkel *et al.* (1985) | 1.23 | 1.87 | 0.81 |
| Geng *et al.* (1988) | 2.16 | 4.29 | 1.08 |
| Humble *et al.* (1987) | 2.34 | 6.75 | 0.81 |
| Inoue & Hirayama (1988) | 2.55 | 8.78 | 0.74 |
| Kabat (1990) | 0.90 | 1.76 | 0.46 |
| Kabat & Wynder (1984) | 0.79 | 2.45 | 0.25 |
| Kalandidi *et al.* (1990) | 1.57 | 2.83 | 0.87 |
| Koo *et al.* (1987) | 1.55 | 2.67 | 0.90 |
| Lam T. *et al.* (1987) | 1.65 | 2.35 | 1.16 |
| Lam W. *et al.* (1985) | 2.01 | 3.72 | 1.09 |
| Lee *et al.* (1986) | 1.03 | 2.55 | 0.41 |
| Liu *et al.* (1991) | 0.74 | 1.69 | 0.32 |
| Pershagen *et al.* (1987) | 1.27 | 2.15 | 0.75 |
| Sobue *et al.* (1990) | 1.31 | 1.96 | 0.87 |
| Svensson *et al.* (1989) | 1.26 | 2.81 | 0.57 |
| Trichopoulos *et al.* (1983) | 2.13 | 3.83 | 1.19 |
| Varela (1987) | 0.87 | 1.09 | 0.69 |
| Wu *et al.* (1985) | 1.41 | 3.67 | 0.54 |
| Wu-Williams *et al.* (1990) | 0.79 | 1.02 | 0.62 |
| Combined analysis | 1.17 | 1.28 | 1.06 |

more likely to be assessed as exposed to ETS: and thus the estimate of relative risk of exposure to ETS will be exaggerated, due to the association of lung cancer with active smoking for this group of 'deceivers'.

The EPA Draft Review (1990) estimated this 'spurious excess risk' to be approximately 0.12, and adjusted their combined risk downward by this amount. We have argued elsewhere (Tweedie *et al.* (submitted)) that the true extent of this misclassification bias is actually somewhat higher than this, and Lee (1988) has an extensive discussion of the basis for establishing an appropriate value. Lee (1991*a*), using the ratio of relative risks for ETS and active smokers, asserts that a more extensive misclassification has also taken place, although Wald *et al.* (1991*a*,*b*) dispute this.

If we accept the (possibly conservative) EPA figure then we would adjust downwards the observed value of 1.17 from the combined analysis to a 'true' underlying value of $RR_{ETS}$ = 1.05.

A second source of potential bias in the other direction is the possibility that the so-called 'unexposed' group is in fact exposed to a certain amount of 'background' ETS. Such background exposure might result in a spuriously *low* value, in contrast to the misclassification effect above. Wald *et al.* (1986), the NRC Report (1986), and the EPA Draft Review (1990) all use the following cotinine argument identical in type to that in Section 2 to deduce the 'true' excess risk due to ETS exposure, over the risk for a hypothetically completely unexposed group.

Suppose levels of cotinine are in principle known for non-smokers exposed to ETS (defined as those with smoking spouses) and for non-smokers supposedly unexposed.

If $\delta$ is the level of cotinine in the supposedly unexposed individuals, if $\alpha$ is the level in supposed non-smokers exposed to ETS, and if $RR_O$ is the *observed* relative risk for exposure to ETS as found from epidemiological studies, then one may find the true relative risk $RR_{ETS} = 1 + \alpha$ from solving

$$RR_O = (1 + \alpha)/(1 + \delta)$$

Wald *et al.* (1986) and the NRC Report (1986), based on data of Wald and Ritchie (1984), adopted the ratio $(\alpha/\delta) = 3.0$ in carrying out this analysis. The value of 3.0 is supported to a certain extent in other literature (see 4-28 of the EPA Draft Review (1990), which justifies and adopts the value of 3.0, and also Table II of Jarvis *et al.* (1984), which indicate values between 2.25 and 4 as reasonable for this ratio).

If we assume there is such a 3-fold larger excess risk for exposed non-smokers, then the real excess risks are given by finding $3\delta$ from

$$RR_O = (1 + 3\delta)/(1 + \delta)$$

If we use the value of 1.05 for $RR_O$ estimated above, then we estimate a relative risk after adjustment on this basis of $RR_{ETS} = 1.076$.

This is of course virtually identical with that found in Section 2 from the dosimetric approach, and thus on the basis of the most commonly acceptable data the two methods appear to be essentially reconciled.

### 4. Methodological questions with meta-analysis

### *Inclusion of studies*

The question of which studies to include in a meta-analysis is a difficult one. Theoretically meta-analysis is valid only insofar as one is combining comparable 'experiments', and in particular comparable exposures, and there is good reason to believe that whatever 'ETS' actually is, it is variable across countries due not only to different chemical composition of tobacco smokes but also widely differing social customs and corresponding levels of exposure.

One could argue that studies from China should not be included: tobacco smoking reportedly occurred on a wide scale more recently there, and this may account for low values such as in the recent studies of Liu *et al.* (1991) or Wu-Williams *et al.* (1991). Clearly these recent results account for part of the lowering in our current estimate from those in 1986 or even 1990. But equally one would then have to consider omitting Geng *et al.* (1988) or Lam (1987) or Lam (1985) on such an argument; and these, conversely, provide much of the increased overall relative risk.

A separate analysis of all US studies in Table I gives an estimated relative risk of 1.10 with a 95% CI of (0.95, 1.27). This might be a more conservative and safer estimate of the level of association of ETS and lung cancer under western conditions, although applying the Wald-EPA correction of 0.12 to this value actually reduces it below unity.

It has also been suggested that inclusion of the Varela (1987) results, because they are only in thesis form and relatively hard to access, is invalid. This seems an extremely dangerous approach to meta-analysis. We have included the results of Geng *et al.* (1988) despite the poor information of the study available; the Varela (1987) study is (once located) particularly well-documented in comparison to most published studies, as it must be to be reviewed by thesis examiners.

The real danger with any meta-analysis is the spurious inflation of results due to omission of unpublished negative results (see Chalmers & Buyse, 1988). One must include, at least initially, all available studies unless there is clear and documented reason to omit them, and the analysis above does this.

### *Use of adjusted estimates*

There are many potential confounders of the relationship between ETS and lung cancer: age, diet, occupation, have all been suggested as possible variables for which results should be adjusted. Use of such adjusted estimates in a meta-analysis is usually not possible, since it confuses the relative risks being combined unless the covariates used in adjustment are the same across studies. In fact this is far from the case, as is shown in Table II which gives odds ratios from raw data, and published figures given as 'adjusted' in various ways for covariates.

These adjustments, when given in the literature, have gone

**Table II** Odds ratios and published 'adjusted' estimates of relative risk

| | *Original* | *Adjusted* | *Difference* | *Factors used* |
|---|---|---|---|---|
| Humble *et al.* (1987) | 2.34 | 1.20 | −1.14 | Age, ethnicity, sex |
| Sobue *et al.* (1990) | 1.31 | 0.94 | −0.37 | Age |
| Koo *et al.* (1987) | 1.55 | 1.19 | −0.36 | Age, parity, education, years since exposure ceased |
| Wu *et al.* (1985) | 1.41 | 1.20 | −0.21 | Age, active smoking |
| Wu-Williams *et al.* (1990) | 0.79 | 0.70 | −0.09 | Age, education, centre |
| Lee *et al.* (1986) | 1.03 | 1.00 | −0.03 | Age |
| Liu *et al.* (1991) | 0.74 | 0.77 | 0.03 | Age, education, area |
| Varela (1987) | 0.87 | 0.94 | 0.07 | Age, sex, residence, etc |
| Brownson *et al.* (1987) | 1.52 | 1.68 | 0.16 | Age |
| Kalandidi *et al.* (1990) | 1.57 | 1.92 | 0.35 | Age, education, energy intake, diet, interviewer |
| Garfinkel *et al.* (1985) | 1.23 | 1.70 | 0.47 | Age, hospital |
| Gao *et al.* (1987) | 1.19 | 1.70 | 0.51 | Age, education |
| Inoue & Hirayama (1988) | 2.55 | 3.09 | 0.54 | Age, year of death, hospital |
| Lam *et al.* (1985) | 2.01 | 2.64 | 0.63 | Not known |
| Svensson *et al.* (1989) | 1.26 | 1.90 | 0.64 | Age |
| Pershagen *et al.* (1987) | 1.27 | 2.40 | 1.13 | Age |

either way. In the recent work of Sobue *et al.* (1990), the adjustment provides an estimate of 0.94 compared with a raw figure of 1.31; the estimates of Koo *et al.* (1987) and Wu-Williams *et al.* (1990), on quite large studies, similarly drop after adjustment for covariates; and conversely, the values of Garfinkel *et al.* (1985), Gao *et al.* (1987) and Pershagen *et al.* (1987) show increases in large studies.

The very obvious effects of confounding that the adjusted estimates in Table II indicate must be a cause of concern. Overall, therefore, we have chosen as have all other reviews to work with the raw data.

We have also not included the three existing cohort studies of Hirayama (1981; 1984), Gillis *et al.* (1984) or Garfinkel (1981). The Gillis study is too small to influence the results. With the other two major studies, there is a substantial question as to whether to use raw data or age-adjusted data. The age-adjusted results in Garfinkel (1981) are 1.18 with a CI of (0.90, 1.54), a huge increase over the raw result of 0.53 with a 95% CI of (0.38, 0.73). There are a number of ways of age-adjusting the Hirayama data (cf Ahlborn & Uberla, 1988; Tweedie (submitted)), and the latter version of this gives a value of 1.15 as opposed to the value for non-adjusted figures of 1.36 with a 95% CI of (0.95, 1.96).

These are wide variations, and as with the case-control studies in Table II, age-adjustment does not give uniform change. For the same reasons that we have avoided the adjusted figures of Table II we have therefore omitted them here, although this is not material as the age-adjusted values do not alter the overall conclusion of our analysis.

*Levels of significance*

The inclusion of new studies has changed the point estimate from the combined analysis from 1.42 in the EPA Draft Review (1990) to 1.17, although this is still, on the basis of the variance from the combined analysis, an estimate which is significant at over the 99% level.

As noted in the EPA Draft Review (1990), one can also carry out a non-parametric test of the significance of the data. Figure 1 shows these odds ratios in descending order, with the 95% confidence intervals from Table I. The sign test on these studies also indicates that, since only seven of 26 studies give values below 1.00, there is also a significant rejection of the hypothesis of no association at the 99% level on this non-parametric basis.

Moreover, as in previous studies we find a value from the combined analysis which is still noticeably (if no longer significantly) above that from the most recent evaluations of biochemical marker data.

However, as observed by Wald *et al.* (1986), this significance may be due to differential misclassification, and the adjustments in the previous section take account of this. We

**Figure 1** Relative risks of case control studies of females.

must then make a corresponding adjustment of the confidence interval in this method. The standard approach has been to adjust the confidence limits themselves by the same amount, i.e. to propose a 95% CI of (0.92, 1.14) based on Table I. This is almost certainly tighter than is warranted, since many of the adjusting figures are themselves open to error: see Mantel (1990). Indeed, one could well use the results of recent studies, and the range of risks reported, as support for the view that the previously reported results were less accurate than the published CIs would indicate. However, even at these levels the meta-analysis shows the current $RR_{ETS}$ to be insignificantly raised at the 5% level. If the correction of 0.12 is applied to the individual estimates, the adjustment to Figure 1 shows this also implies that the number of positive results is no longer significant at the 95% level using the sign test, whilst changing the individual confidence intervals in this way leaves only 7.5% of the results significant at the 5% level, which is also very consistent with a random effect.

There is clearly more work needed to develop statistical methods for analysing this type of adjustment method, since the interpretation of the statistical significance of the adjusted data is now at a very subjective level.

### 5. Discussion

This paper addresses two questions of on-going importance, not only in the assessment of the relationship between ETS and occurrence of lung cancer, but also in the methodology of evaluation of results which are inherently highly variable and hard to assess.

The first is in the actual level of relative risk to be ascribed to ETS. It has been argued (Wald *et al.*, 1991*b*) that '. . . passive smoking is a low dose exposure to a mixture of substances that, at a high dose, is one of the best documented and most potent causes of human cancer.' This biological plausibility is of course the rationale for seeking to assess, by a number of means, whether the exposure incurred by non-smokers does lead to a substantially increased relative risk, and if so what the level is. It has therefore caused some considerable confusion when two apparently valid approaches to the problem give results which have seemingly differed by an order of magnitude, and where to date the last word has been the somewhat pessimistic statement by Wald *et al.* (1991*b*) that it is '. . . not reasonable to expect a close quantitative consistency . . .' between the two, and that given the uncertainties in both methods '. . . it is remarkable that the cotinine levels and the risk estimate [from epidemiological studies] are as concordant as they are'.

There are of course very many difficulties and assumptions in the meta-analysis method, especially in the combining of epidemiological data. It can be criticised because of the differences in the studies that are being put together: there is no clear reason to believe that exposure to ETS in the United Sates is the same exposure as in Asia, for example, nor that the exposure for males is the same as for females. There is also the possibility that negative studies may be omitted (Chalmers, 1988), leading to an overestimate based on selective inclusion of positive studies and omission of negative studies. There are well-accepted biases due to misclassification in individual studies, and although the Wald technique in Section 3 is now used routinely to adjust for these, it can still be argued (cf Lee, 1991*a,b*) that the study results are even more biased and the corresponding meta-analysis estimate is too high.

But there are also difficulties and assumptions in the biological marker method. The argument assumes that excess risk is linearly related to cotinine levels: this both assumes that cotinine is an accurate quantitative measure of carcinogen intake if any, and moreover that it is linearly related to the results of such carcinogen intake. It also relies on the *current* levels of cotinine (which indicate nicotine intake over the past 2–5 days) as being accurate estimates of the background and foreground exposure in the never-smokers over their whole past history. Given that lung cancers are not a product of short term exposures, this entails a major assumption about the relative risks, and stability of those risks, over long time periods.

Repace and Lowry (1990, Section 3) provide a good discussion of the benefits and dangers inherent in using cotinine to evaluate risk, but conclude that several authorities have found it an 'adequate basis for exposure assessment purposes'.

It is somewhat surprising, then, that studies such as the NRC Report (1986) or Repace and Lowry (1990) adopt the epidemiologically based value for the overall relative risk to females for exposure to ETS, rather than the values derived from cotinine arguments.

The arguments for its rejection seem to be rather flimsy. Darby and Pike (1989), p. 338) feel that 'it may simply be that cotinine is not an adequate measure of the exposure of non-smokers to the carcinogenic components of cigarette smoke'. Repace and Lowry (1990, p. 31) state that the 'exposure-response relationship was found to be inconsistent with the epidemiology of passive smoking, and was abandoned' in favour of other phenomenological approaches.

Despite this rejection of the cotinine argument for assessing the overall relative risk of exposure to ETS, it nonetheless continues to be used to make major adjustments for 'background exposure' bias in the review papers cited above, with an increase of virtually 50% in excess risk estimates based on the biochemical argument marker as in Section 3. The logic for this acceptance of cotinine in one part of the analysis and its rejection in another is hard to support.

In this paper we have shown that, using the current published studies of lung cancer and its association with exposure to ETS, there is no need to reject either approach because of the difference in results found.

We have shown that the range of estimates of 1.03–1.10 for the relative risk for exposure to ETS, as calculated by the biological marker method in Section 2, is almost identical with the best estimates from combined analysis of current epidemiological studies given in Section 3, provided one accepts the level of bias indicated for the case-control models in the EPA Draft Review (1990), and makes a consistent use of the cotinine argument to support a 50% increase in the estimate by taking into account bias from background exposure.

This result is at variance with those in the reviews to date, and is some five to 10-fold weaker than the estimate in the EPA Draft Review (1990). The methodology here is identical: the only difference is in the addition of a number of new studies.

What could cause such a change in estimates? Formally, it is because the more recent studies include some large (and hence presumed by the methodology to be more reliable) recent studies with estimates of relative risk below the previous combined estimate. Indeed, a considerable amount of the change is due to the inclusion of the Kabat (1990), Varela (1987), and Wu-Williams (1990) studies, although in the other direction the Sobue *et al.* (1990) and Fontham (1991) studies provide a higher estimate.

Such oscillating outcomes can be confusing, and meta-analysis properly used should enable a clearer overview of the true picture. The second contribution of this paper is to indicate the caution that must be used in setting up such analyses, and to caution against over-interpreting the point estimates from the meta-analysis method. Our point estimate (prior to adjustment for the two types of misclassification) is 1.17 and this seems much lower than the 1.42 of the EPA Draft Report (1990). But the 95% confidence intervals for these estimates are (1.06, 1.28) and (1.24, 1.63) respectively, and although this indicates that the results of new studies are overall below what might have been expected, they are certainly not giving a total different picture.

More substantially, however, the analysis we give shows clearly the need to include all studies to gain an accurate picture of the overall risk ratio.

There are still some serious methodological questions about meta-analysis in the presence of such a difficult to measure exposure as ETS, especially in the presence of differential misclassification. Mantel (1990) has argued that the very low levels of excess risk reported in studies of ETS and lung cancer are in the region where epidemiological studies can never establish excess risk estimates significantly above zero. Certainly, the contribution of random error at the current state of analysis is not at all clear.

For this reason it is important to develop other techniques which might help establish a figure with greater accuracy. More and better controlled studies help to do this, but so do indirect inferential methods such as the cotinine marker approach. It is therefore of considerable value that the different approaches appear to be reconcilable, rather than leaving open a subjective judgement of which to accept.

The comments of Professors John Eccleston and Ian Saunders, and the input from referees, have sharpened considerably our treatment on the potential pitfalls with the meta-analysis method.

This work was initiated whilst both authors were at the School of Information and Computing Sciences, Bond University, Australia.

**References**

AHBORN, W. & UBERLA, K. (1988). Passive smoking and lung cancer: reanalyses of Hirayama's data. *Indoor and Ambient Air Quality*, R. Perry, (ed.) London.

AKIBA, S., KATO, H. & BLOT, W.J. (1986). Exposure to ETS and lung cancer among Japanese women. *Cancer Res.*, **46**, 4804- 4807.

BROWNSON, R.C., REIF, J.S., KEEFE, T.J., FERGUSON, S.W. & PRITZL, J.A. (1987). Risk factors for adenocarcinoma of the lung. *Am. J. Epidemiol.*, **125**, 25–34.

BUFFLER, P.A., PICKLE, L.W., MASON, T.J. & CONSTANT, C. (1984). The causes of lung cancer in Texas. In: *Lung Cancer: Causes and Prevention*. Mizell, M. & Correa, P. (eds). Verlag Chemie International: New York, pp. 83–89.

CHALMERS, T.C. & BUYSE, M.E. (1988). Meta-analysis. In *Data Analysis for Clinical Medicine*, Chalmers, T.C., (ed.). pp. 75–85. International Uni Press, Rome.

CHAN, W.C. & FUNG, S.C. (1982). Lung cancer in non-smokers in Hong Kong. *Cancer Campaign*, Vol. 6, Cancer Epidemiology, pp. 199–202.

CORREA, P., PICKLE, L.W., FONTHAM, E.L., LIN, Y. & HAENSZEL, W. (1983). Exposure to ETS and Lung Cancer. *Lancet*, **ii**, 595–597.

DARBY, S.C. & PIKE, M.C. (1988). Lung cancer and passive smoking: predicted effects from a mathematical model for cigarette smoking and lung cancer. *Br. J. Cancer*, **58**, 825–831.

EPA DRAFT REVIEW (1990). Health effects of passive smoking: assessment of lung cancer in adults and respiratory disorders in children. United States EPA, Washington.

FONTHAM, E.T.H., CORREA, P., WU-WILLIAMS, A., REYNOLDS, P., GREENBERG, A.S., BUFFLER, P.A., CHEN, V.W., BOYD, P., ALTERMAN, T., AUSTIN, D.F., LIFF, J. & GREENBERG, S.D. (1991). Cancer in non-smoking women: a multicenter case-control study. *Cancer Epidemiol. Biomarkers & Prevention*, **1**, 35–43.

GAO, Y.-T., BLOT, W.J., ZHENG, W., ERSHOW, A.G., HSU, C.W., LEVIN, L.I., ZHANG, R. & FRAUMENI, J.F. (1987). Lung cancer among Chinese women. *Int. J. Cancer*, **40**, 604–609.

GARFINKEL, L. (1981). Time trends in lung cancer mortality among non-smokers and a note on exposure to ETS. *JNCI*, **66**, 1061–1066.

GARFINKEL, L., AUERBACH, O. & JOUBERT, L. (1985). Involuntary smoking and lung cancer: a case study, *JNCI*, **75**, 463–469.

GENG, G.-Y., LIANG, Z.H., ZHANG, A.Y. & WU, G.L. (1988). On the relationship between smoking and female lung cancer. In Aoki, M., Hisamichi, S. & Tominaga, S. (eds), *Smoking and Health 1987*, Proceedings of the 6th World Conference on Smoking and Health, Tokyo.

GILLIS, C.R., HOLE, D.J., HAWTHORNE, V.M. & BOYLE, P. (1984). The effect of environmental tobacco smoke in two urban communities in the West of Scotland. *Eur. J. Resp. Dis. (Suppl)*, **133**, 121–126.

HIRAYAMA, T. (1981). Non-smoking wives of heavy smokers have a higher risk of lung cancer: a study from Japan. *Br. Med. J.*, **282**, 183–185.

HIRAYAMA, T. (1984). Lung cancer in Japan: effects of nutrition and exposure to ETS. In *Lung Cancer: Causes and Preventions*. Verlag Chemie Weinheim, pp. 175–195.

HUMBLE, C.G., SAMET, J.M. & PATHAK, D.R. (1987). Marriage to a smoker and lung cancer risk. *Am. J. Public Health*, **77**, 598–602.

INOUE, R. & HIRAYAMA, T. (1988). Passive smoking and lung cancer in women. In Aoki, M., Hisamichi, A. & Tominaga, S. (eds), *Smoking and Health 1987*, Proceedings of the 6th World Conference on Smoking and Health, Tokyo.

JARVIS, M., TUNSTALL-PEDOE, H., FEYERABEND, C., VESEY, C. & SALLOOJEE, Y. (1984). Biochemical markers of smoke absorption and self-reported exposure to ETS. *J. Epid. Comm. Health*, **38**, 335–339.

KABAT, G.C. (1990). Epidemiologic studies of the relationship between passive smoking and lung cancer. *Proc 1990 Toxicology Forum*, 187–199.

KABAT, G.C. & WYNDER, E.L. (1984). Lung cancer in nonsmokers. *Cancer*, **53**, 1214–1221.

KALANDIDI, A., KATSOUYANNI, K., VOROPOULOU, N., BASTAS, G., SARACCI, R. & TRICHOPOULOS, D. (1990). Passive smoking and diet in the aetiology of lung cancer among non-smokers. *Cancer Causes and Control*, **1**, 15–21.

KOO, L.C., HO, J.H., SAW, D. & HO, C. (1987). Measurement of passive smoking and estimates of lung cancer risk among non-smoking Chinese females. *Int. J. Cancer*, **39**, 162–169.

LAM, T.H., KUNG, I.T.M., WONG, C.M., LAM, W.K., KLEEVENS, J.W.L., SAW, D., HSU, C., SENIVERATNE, S., LAM, S.Y., LO, K.K. & CHAN, W.C. (1987). Smoking, passive smoking and histological types in lung cancer in Hong Kong Chinese women. *Br. J. Cancer*, **6**, 673–678.

LAM, W.K. (1985). *A Clinical and Epidemiological Study of Carcinoma of Lung in Hong Kong*, M.D. Thesis submitted to University of Hong Kong..

LEE, P.N., CHAMBERLAIN, J. & ALDERSON, M.R. (1986). Relationship of passive smoking to risk of lung cancer and other smoking-associated diseases. *Br. J. Cancer*, **54**, 97–105.

LEE, P.N. (1988). *Misclassification of Smoking Habits and Passive Smoking: a Review of the Evidence*. Springer-Verlag Berlin.

LEE, P.N. (1991*a*). Lung cancer and passive smoking: letter to the editor. *Br. J. Cancer*, **63**, 161–162.

LEE, P.N. (1991*b*). Lung cancer and passive smoking (continued): letter to the editor. *Br. J. Cancer*, **64**, 200.

LIU, Z., HE, X. & CHAPMAN, R.S. (1991). Smoking and other risk factors for lung cancer in Xuanwei, China. *Int. J. Epidemiol.*, **20**, 26–31.

MANTEL, N. (1990). What is the epidemiological evidence for a passive smoking - lung cancer association? In *Indoor Air Quality*, H. Kasuga, (ed.) Springer-Verlag, Berlin, pp. 341–347.

NRC COMMITTEE ON PASSIVE SMOKING (1986). *Environmental Tobacco Smoke – Measuring Exposures and Assessing Health Effects*. National Academy Press, Washington.

PERSHAGEN, G., HRUBEC, Z. & SVENSSON, C. (1987). Passive smoking and lung cancer in Swedish women. *Am. J. Epidem.*, **125**, 17–24.

REPACE, J.L. & LOWREY, A.H. (1990). Risk assessment methodologies for passive smoking-induced lung cancer. *Risk Anal.*, **10**, 27–37.

SOBUE, T., SUZUKI, R., NAKAYAMA, N. & 14 others (1990). Passive smoking among non-smoking women and the relationship between indoor air pollution and lung cancer incidence – results of a multicentre case controlled study. *Gan to Rinsho*, **36**, 329–332.

SVENSSON, C., PERSHAGEN, G. & KLOMINEK, J. (1989). Smoking and passive smoking in relation to lung cancer in women. *Acta Oncol.*, **28**, 623–629.

TRICHOPOULOS, D., KALANDIDI, A., SPARROS, L. & MACMAHON, B. (1981). Lung cancer and exposure to ETS. *Int. J. Cancer*, **27**, 1–4.

TRICHOPOULOS, D., KALANDIDI, A. & SPARROS, L. (1983). Lung cancer and exposure to ETS. Conclusion of Greek study. *Lancet*, **ii**, 677–678.

TWEEDIE, R.L., MENGERSEN, K.L. & ECCLESTON, J.A. (1992). Confounding and misclassification effects in case-control studies of lung cancer incidence (submitted).

TWEEDIE, R.L. (1992). Age-adjustment in passive smoking studies (submitted).

UBERLA, K. (1987). Cancer from passive smoking: hypothesis or convincing evidence? *Int. Arch. Occup. Environ. Health*, **59**, 421–437.

VARELA, L.R. (1987). *Assessment of the Association Between Passive Smoking and Lung Cancer*, PhD Thesis, Yale University.

VUTUC, C. (1984). Quantitative aspects of passive smoking and lung cancer. *Prev. Med.*, **13**, 698–704.

WALD, N.J. & RITCHIE, C. (1984). Validation of studies on lung cancer in non-smokers married to smokers. *Lancet*, **i**, 1067.

WALD, N.J., NANCHAHAL, K., THOMPSON, S.G. & CUCKLE, H.S. (1986). Does breathing other people's tobacco smoke cause lung cancer? *Br. Med. J.*, **293**, 1217–1222.

WALD, N.J., NANCHAHAL, K., CUCKLE, H.S. & THOMPSON, S.G. (1990). Lung cancer and passive smoking: letter to the editor. *Br. J. Cancer*, **61**, 337.

WALD, N.J., CUCKLE, H.S., NANCHAHAL, K. & THOMPSON, S.G. (1991*a*). Response to the Letter from Dr P. Lee: Letter to the editor. *Br. J. Cancer*, **63**, 163.

WALD, N.J., CUCKLE, H.S., NANCHAHAL, K. & THOMPSON, S.G. (1991*b*). Response to letter from Dr Lee: Letter to the editor. *Br. J. Cancer*, **64**, 201.

WU, A.H., HENDERSON, B.E., PIKE, M.C. & YU, M.C. (1985). Smoking and other risk factors for lung cancer in women. *JNCI*, **74**, 747–751.

WU-WILLIAMS, A.H. & SAMET, J.M. (1990). Environmental tobacco smoke: exposure-response relationships in epidemiologic studies. *Risk Anal.*, **10**, 39–48.

WU-WILLIAMS, A.H., DAI, X.D., BLOT, W., XU, Z.Y., SUN, X.W., XIAO, H.P., STONE, B.J., YU, S.F., FENG, Y.P., ERSHOW, A.G., SUN, J., FRAUMENTI, J.F. Jr & HENDERSON, B.E. (1990). Lung cancer among women in north-east China. *Br. J. Cancer*, **62**, 982–987.

YUSUF, S., PETO, R., LEWIS, J., COLLINS, R. & SLEIGHT, P. (1985). Beta-blockade during and after myocardial infarction: an overview of the randomized trials. *Prog. Cardiovasc. Dis.*, **27**, 335–371.


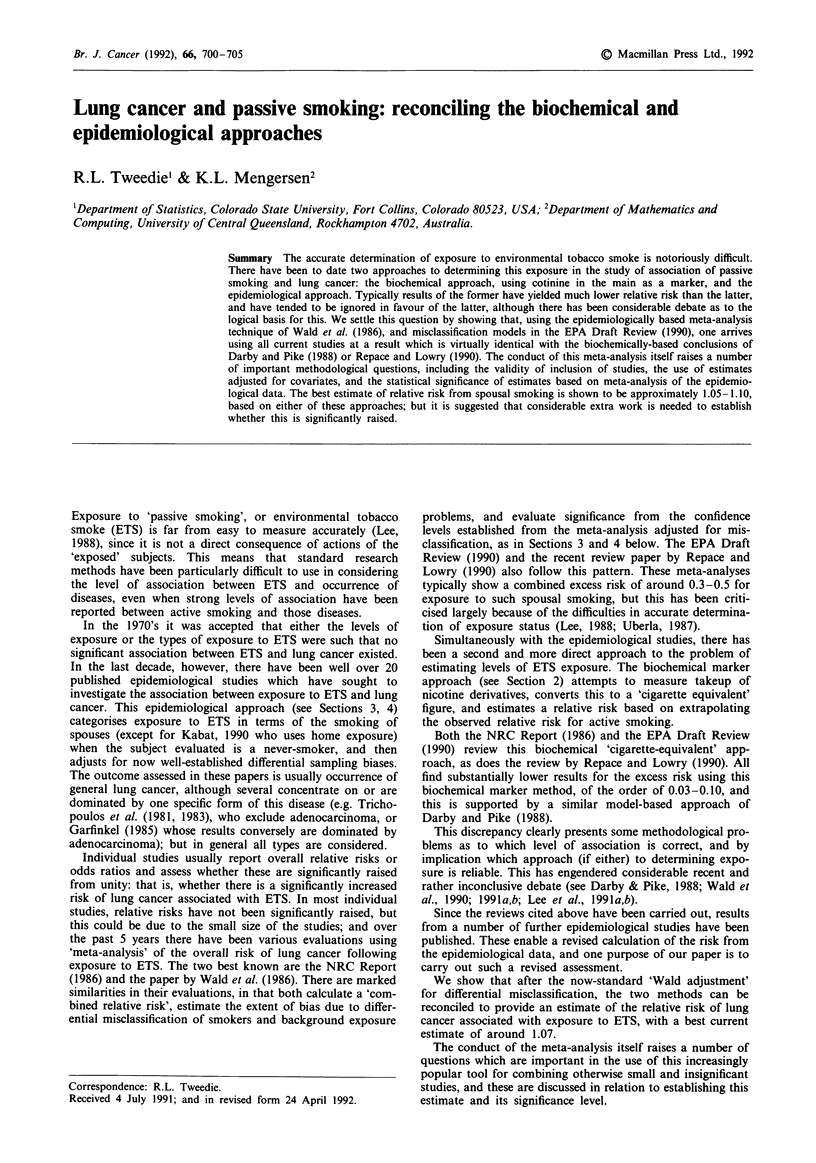

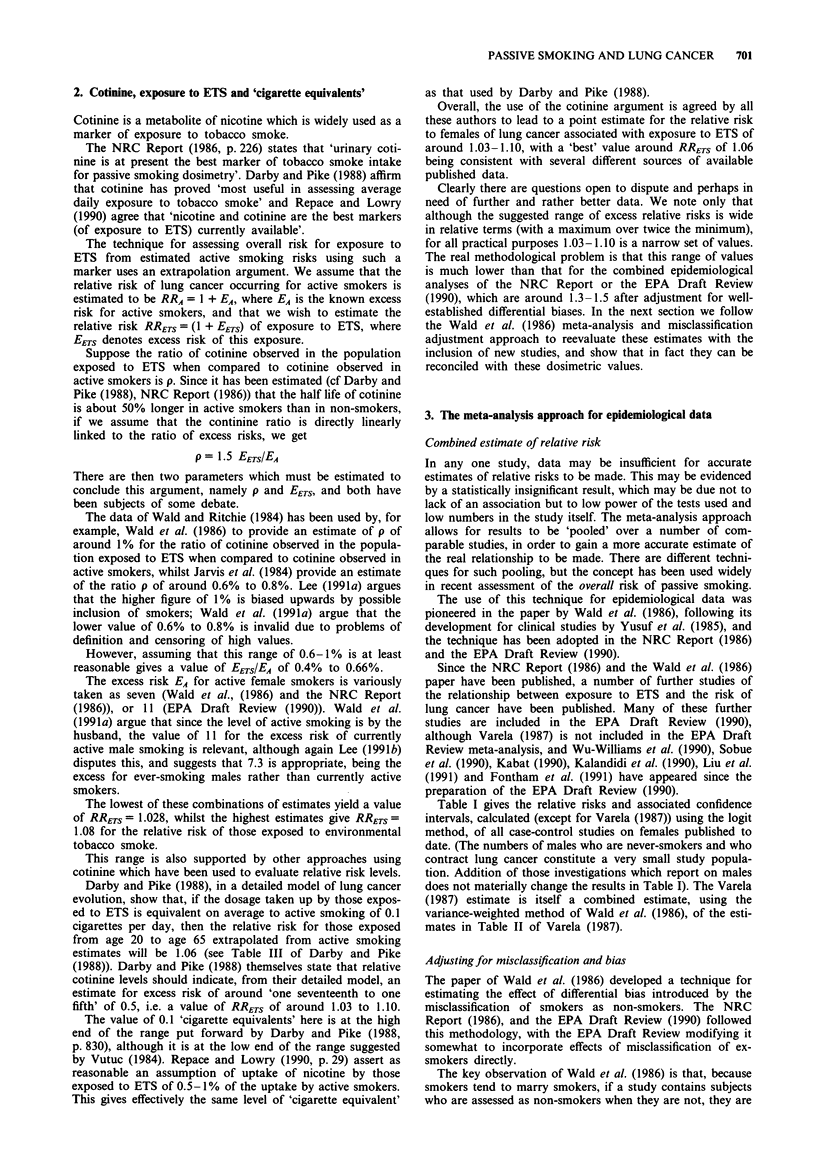

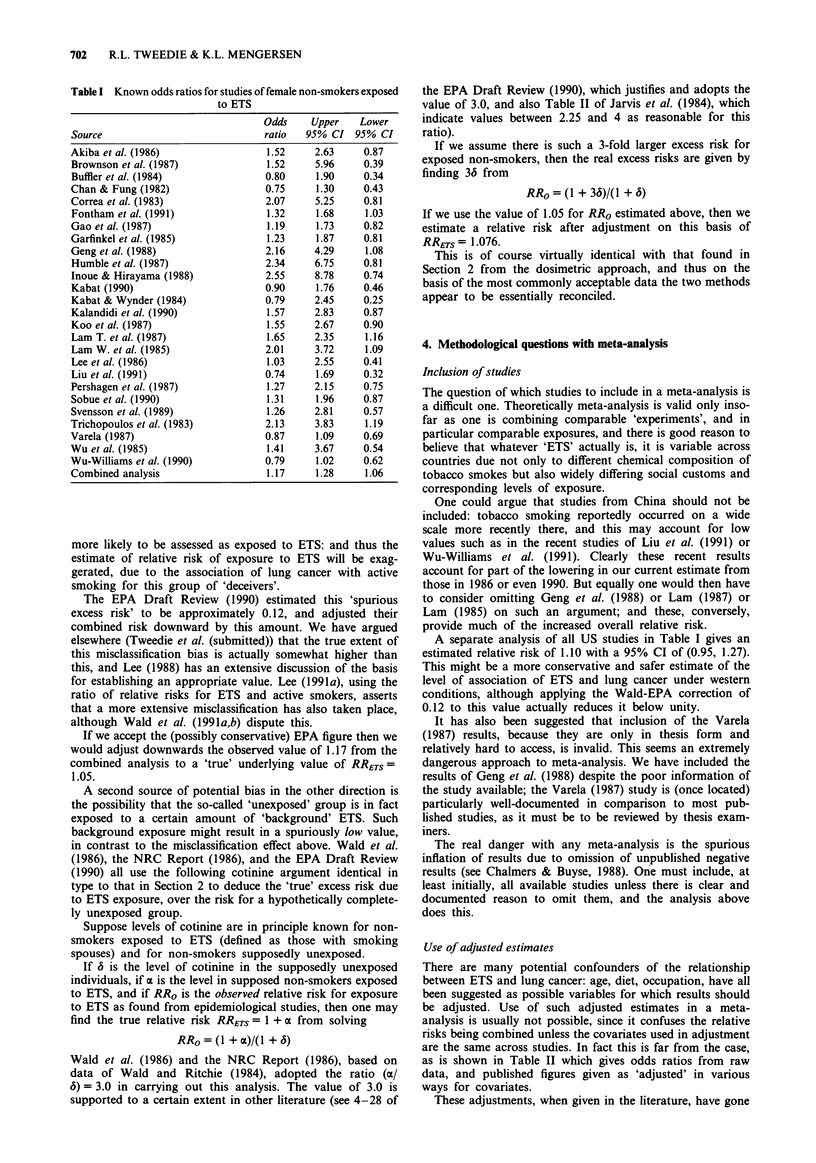

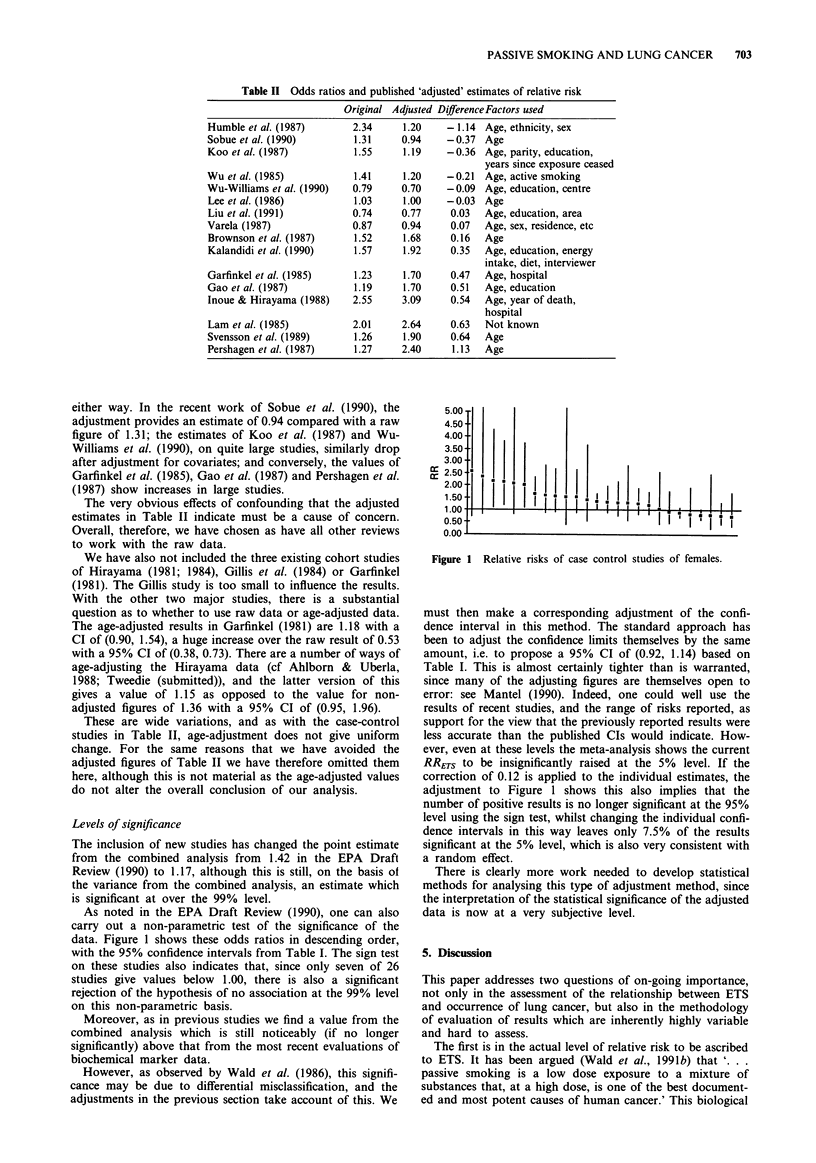

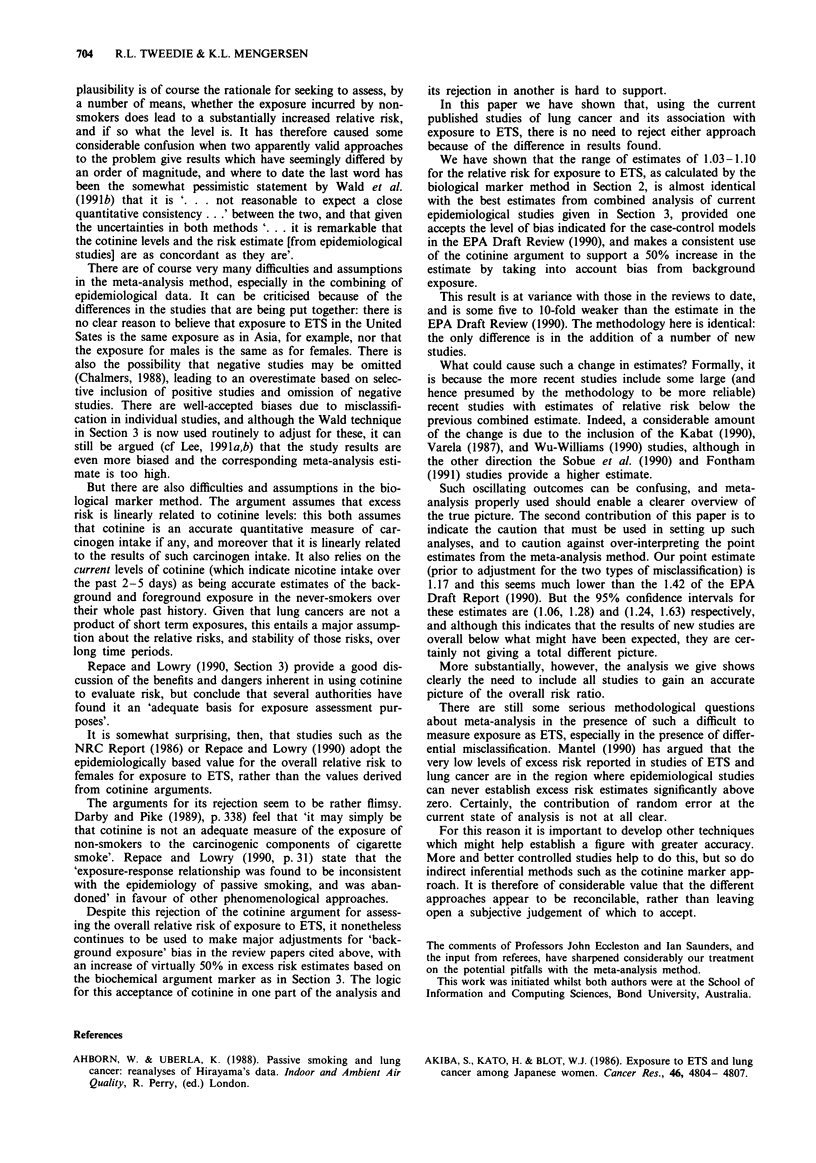

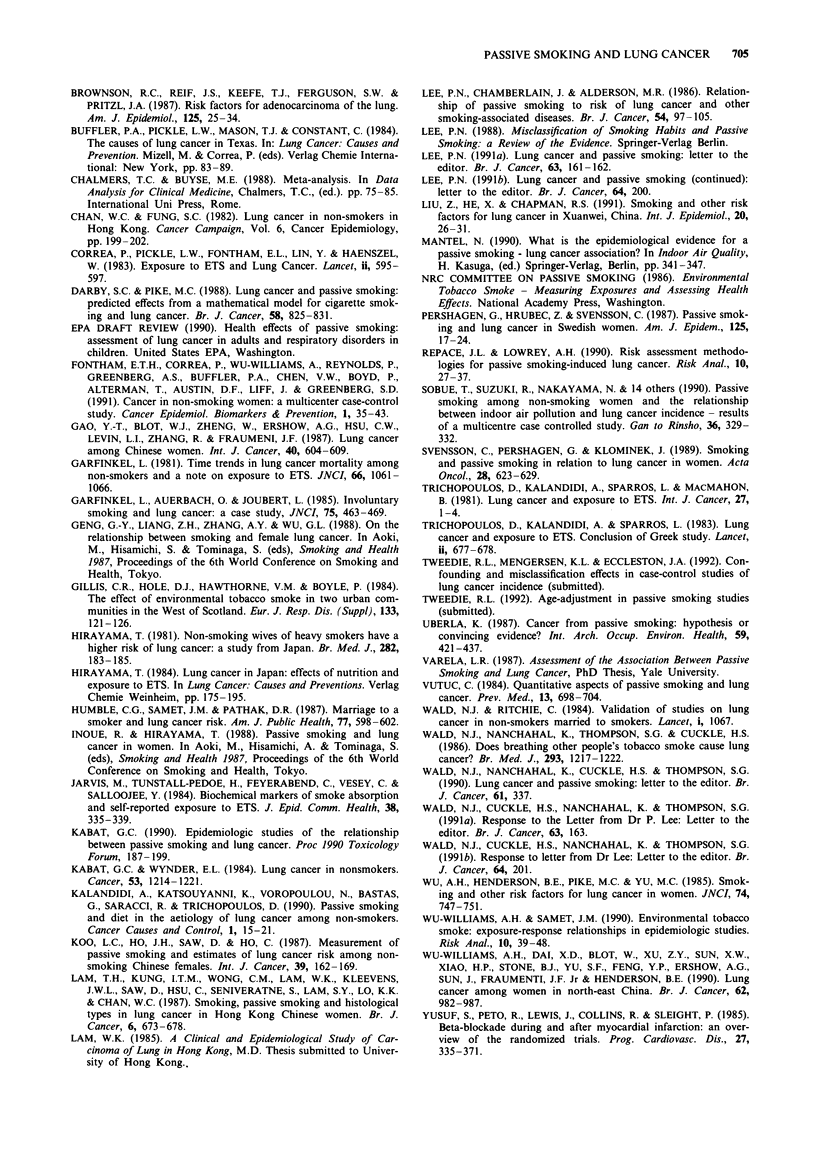


## References

[OCR_00792] Akiba S., Kato H., Blot W. J. (1986). Passive smoking and lung cancer among Japanese women.. Cancer Res.

[OCR_00798] Brownson R. C., Reif J. S., Keefe T. J., Ferguson S. W., Pritzl J. A. (1987). Risk factors for adenocarcinoma of the lung.. Am J Epidemiol.

[OCR_00819] Correa P., Pickle L. W., Fontham E., Lin Y., Haenszel W. (1983). Passive smoking and lung cancer.. Lancet.

[OCR_00824] Darby S. C., Pike M. C. (1988). Lung cancer and passive smoking: predicted effects from a mathematical model for cigarette smoking and lung cancer.. Br J Cancer.

[OCR_00834] Fontham E. T., Correa P., WuWilliams A., Reynolds P., Greenberg R. S., Buffler P. A., Chen V. W., Boyd P., Alterman T., Austin D. F. (1991). Lung cancer in nonsmoking women: a multicenter case-control study.. Cancer Epidemiol Biomarkers Prev.

[OCR_00841] Gao Y. T., Blot W. J., Zheng W., Ershow A. G., Hsu C. W., Levin L. I., Zhang R., Fraumeni J. F. (1987). Lung cancer among Chinese women.. Int J Cancer.

[OCR_00851] Garfinkel L., Auerbach O., Joubert L. (1985). Involuntary smoking and lung cancer: a case-control study.. J Natl Cancer Inst.

[OCR_00846] Garfinkel L. (1981). Time trends in lung cancer mortality among nonsmokers and a note on passive smoking.. J Natl Cancer Inst.

[OCR_00862] Gillis C. R., Hole D. J., Hawthorne V. M., Boyle P. (1984). The effect of environmental tobacco smoke in two urban communities in the west of Scotland.. Eur J Respir Dis Suppl.

[OCR_00868] Hirayama T. (1981). Non-smoking wives of heavy smokers have a higher risk of lung cancer: a study from Japan.. Br Med J (Clin Res Ed).

[OCR_00878] Humble C. G., Samet J. M., Pathak D. R. (1987). Marriage to a smoker and lung cancer risk.. Am J Public Health.

[OCR_00887] Jarvis M., Tunstall-Pedoe H., Feyerabend C., Vesey C., Salloojee Y. (1984). Biochemical markers of smoke absorption and self reported exposure to passive smoking.. J Epidemiol Community Health.

[OCR_00898] Kabat G. C., Wynder E. L. (1984). Lung cancer in nonsmokers.. Cancer.

[OCR_00902] Kalandidi A., Katsouyanni K., Voropoulou N., Bastas G., Saracci R., Trichopoulos D. (1990). Passive smoking and diet in the etiology of lung cancer among non-smokers.. Cancer Causes Control.

[OCR_00908] Koo L. C., Ho J. H., Saw D., Ho C. Y. (1987). Measurements of passive smoking and estimates of lung cancer risk among non-smoking Chinese females.. Int J Cancer.

[OCR_00913] Lam T. H., Kung I. T., Wong C. M., Lam W. K., Kleevens J. W., Saw D., Hsu C., Seneviratne S., Lam S. Y., Lo K. K. (1987). Smoking, passive smoking and histological types in lung cancer in Hong Kong Chinese women.. Br J Cancer.

[OCR_00925] Lee P. N., Chamberlain J., Alderson M. R. (1986). Relationship of passive smoking to risk of lung cancer and other smoking-associated diseases.. Br J Cancer.

[OCR_00938] Lee P. (1991). Lung cancer and passive smoking (continued). Br J Cancer.

[OCR_00934] Lee P. (1991). Lung cancer and passive smoking.. Br J Cancer.

[OCR_00942] Liu Z. Y., He X. Z., Chapman R. S. (1991). Smoking and other risk factors for lung cancer in Xuanwei, China.. Int J Epidemiol.

[OCR_00957] Pershagen G., Hrubec Z., Svensson C. (1987). Passive smoking and lung cancer in Swedish women.. Am J Epidemiol.

[OCR_00962] Repace J. L., Lowrey A. H. (1990). Risk assessment methodologies for passive smoking-induced lung cancer.. Risk Anal.

[OCR_00974] Svensson C., Pershagen G., Klominek J. (1989). Smoking and passive smoking in relation to lung cancer in women.. Acta Oncol.

[OCR_00984] Trichopoulos D., Kalandidi A., Sparros L. (1983). Lung cancer and passive smoking: conclusion of Greek study.. Lancet.

[OCR_00979] Trichopoulos D., Kalandidi A., Sparros L., MacMahon B. (1981). Lung cancer and passive smoking.. Int J Cancer.

[OCR_00998] Uberla K. (1987). Lung cancer from passive smoking: hypothesis or convincing evidence?. Int Arch Occup Environ Health.

[OCR_01007] Vutuc C. (1984). Quantitative aspects of passive smoking and lung cancer.. Prev Med.

[OCR_01015] Wald N. J., Nanchahal K., Thompson S. G., Cuckle H. S. (1986). Does breathing other people's tobacco smoke cause lung cancer?. Br Med J (Clin Res Ed).

[OCR_01020] Wald N., Nanchahal K., Cuckle H., Thompson S. (1990). Lung cancer and passive smoking.. Br J Cancer.

[OCR_01011] Wald N., Ritchie C. (1984). Validation of studies on lung cancer in non-smokers married to smokers.. Lancet.

[OCR_01045] Wu-Williams A. H., Dai X. D., Blot W., Xu Z. Y., Sun X. W., Xiao H. P., Stone B. J., Yu S. F., Feng Y. P., Ershow A. G. (1990). Lung cancer among women in north-east China.. Br J Cancer.

[OCR_01040] Wu-Williams A. H., Samet J. M. (1990). Environmental tobacco smoke: exposure-response relationships in epidemiologic studies.. Risk Anal.

[OCR_01035] Wu A. H., Henderson B. E., Pike M. C., Yu M. C. (1985). Smoking and other risk factors for lung cancer in women.. J Natl Cancer Inst.

[OCR_01052] Yusuf S., Peto R., Lewis J., Collins R., Sleight P. (1985). Beta blockade during and after myocardial infarction: an overview of the randomized trials.. Prog Cardiovasc Dis.

